# Join us on an amazing journey towards next‐generation treatments for CNS disorders: Launch of *Neuroprotection*, a new high‐quality journal in translational neuroscience

**DOI:** 10.1002/nep3.8

**Published:** 2022-09-03

**Authors:** Xunming Ji, Piotr Walczak, Heleen M. M. van Beusekom, Ana I. Casas, Andrew Clarkson, Tracy Farr, Jukka Jolkkonen, Yajie Liang, Michel M. Modo, Paulo H. Rosado‐de‐Castro, Karsten Ruscher, Yan‐Jiang Wang, Haitao Wu, Marietta Zille, Shen Li, Johannes Boltze

**Affiliations:** ^1^ Beijing Institute of Brain Disorders Capital Medical University Beijing China; ^2^ Department of Diagnostic Radiology and Nuclear Medicine, Center for Advanced Imaging Research University of Maryland Baltimore Maryland USA; ^3^ Department of Cardiology, Erasmus MC Rotterdam University Medical Center Rotterdam Rotterdam The Netherlands; ^4^ Department of Neurology, Center for Translational Neuro‐ and Behavioral Sciences (C‐TNBS) University Clinics Essen Essen Germany; ^5^ Department of Anatomy Brain Health Research Center and Brain Research New Zealand Dunedin New Zealand; ^6^ Medical School, Queen's Medical Centre University of Nottingham Nottingham UK; ^7^ A.I. Virtanen Institute for Molecular Sciences University of Eastern Finland Kuopio Finland; ^8^ McGowan Institute for Regenerative Medicine University of Pittsburgh Pittsburgh Pennsylvania USA; ^9^ Department of Radiology University of Pittsburgh Pittsburgh Pennsylvania USA; ^10^ Department of Bioengineering University of Pittsburgh Pittsburgh Pennsylvania USA; ^11^ Department of Anatomy and Department of Radiology, School of Medicine, Institute of Biomedical Sciences Federal University of Rio de Janeiro Rio de Janeiro Brazil; ^12^ Laboratory for Experimental Brain Research University of Lund Lund Sweden; ^13^ Department of Neurology, Daping Hospital Center for Clinical Neuroscience Chongqing China; ^14^ Department of Neurobiology Beijing Institute of Basic Medical Sciences Beijing China; ^15^ Department of Pharmaceutical Sciences, Division of Pharmacology and Toxicology University of Vienna Vienna Austria; ^16^ Department of Neurology and Psychiatry, Beijing Shijitan Hospital Capital Medical University Beijing China; ^17^ School of Life Sciences University of Warwick Coventry UK

**Keywords:** cytoprotection, nervous system repair, neurodegeneration, neuroprotection, neurovasculardiseases, translational research

1

Translational medicine in neurodegenerative and neurovascular diseases is approaching a breakthrough point. Recent years have led to dramatic progress in both experimental and clinical research. Based on a much better and continuously increasing understanding of disease mechanisms, progression and pathophysiology, new therapies with an improved translational potential to protect tissue either against acute or chronic degeneration and even approaches potentially capable of repairing damaged brain tissue are emerging. Importantly, the field has learned from previous translational setbacks as the internal and external validity of experimental studies are continuously improving. This is paralleled by an increase in the number of clinical trials aimed at establishing new therapeutic approaches.

For instance, neuroprotection is currently seeing a renaissance in the areas of stroke and traumatic brain injury research and treatment. The translational failure of early neuroprotective paradigms for stroke which rely on systemic administration of neuroprotectants during acute and subacute stages has been reported and analyzed in detail. Whereas previous preclinical research and the translation of results obtained therein into clinical treatments were often impaired by shortcomings in experimental design, research quality in the field has improved substantially and methodological rigor applied today is outstanding across many fields.^1^ Moreover, the era of recanalization therapies now offers the possibility to apply neuroprotective treatments locally, when and where they are needed.^2^ Moreover, the application strategies for neuroprotectants have been greatly refined. Indeed, contemporary approaches do not focus primarily on permanent neuroprotection trying to rescue larger parts of infarcted brain tissue by targeting single pathomechanisms. Instead, they use elaborate approaches to protect the penumbra in order to gain time for recanalization, to prevent reperfusion injury, or to target different brain resident cells in their respective protective time windows.^3^ Many of these approaches are safe to be applied in a prehospital setting, opening new ways for more targeted and effective ‘2nd generation’ neuroprotective treatment paradigms. Moreover, these new paradigms are developed to be fully compatible with established treatment procedures including recanalization approaches.^4^

Similar progress is reported in other fields. For example, a recent double‐blind and randomized phase II clinical trial in multiple sclerosis (MS) revealed preliminary evidence for a modest yet clinically meaningful therapeutic effect of mesenchymal stem cell (MSC) transplantation.^5^ MSCs can exert therapeutic effects via the secretion of numerous growth factors and have strong immunomodulatory abilities, making them an ideal resource for central nervous system regenerative medicine and beyond. Indeed, MSC transplantation was accompanied by a marked reduction of cerebrospinal fluid levels of neurofilament L, indicating neuronal loss, and CXCL‐13, a potent B‐cell chemoattractant and biomarker for active lesions in MS.

Recent studies have evaluated the therapeutic potential of rasagiline, a monoamine oxidase B inhibitor, regarding its neuroprotective potential in Parkinson's disease (PD) and in amylotrophic lateral sclerosis (ALS). In PD, rasagiline was used as an add‐on to the standard levodopa treatment in a Japanese cohort enrolled into a randomized, double‐blind, placebo‐controlled, Phase II/III clinical trial. Rasagiline reduced OFF‐time and improved symptoms in ON‐time as measured by the Movement Disorder Society‐Unified Parkinson's Disease Rating Scale scores.^6^ A subsequent trial investigating the impact of rasagiline in ALS did not indicate overall efficacy but post‐hoc analyses suggested that rasagiline may provide benefits in some ALS patient subgroups.^7^ To further highlight recent advancements in understanding mechanistic insight, where we once thought monoamine oxidase B played a role in monaime synthesis, we now know that monoamine oxidase B plays a role in astrocytic GABA‐mediated tonic inhibition.^8^ Neuroprotection is also promising and important for the treatment of AD.^9^

Over the past decade, we have experienced an acceleration in the development of new classes of therapeutic agents. While the previous wave of neuroprotective agents in the early 2000s was driven mainly by small molecule drugs, we now live in an age of biopharmaceutical drugs that are beginning to dominate the clinical realm, including antibodies, nanobodies, viral vectors, antisense oligonucleotides, or various gene‐editing solutions. Biopharmaceuticals offer an unprecedented sophistication in therapeutic mechanism and specificity. The challenge, however, is their size, which on average exceeds the size of small molecules by at least three orders of magnitude. The blood–brain barrier is a major obstacle and there is still an unmet need to deliver these very large biologics to reach their targets in the brain. To address this, dedicated delivery techniques such as invasive but highly effective convection‐enhanced delivery^10^ or a minimally invasive intra‐arterial approach^11^ will be required.

Indeed, suboptimal drug access to the brain after systemic administration is believed to be one of the major reasons for the failure of all previous neuroprotective clinical trials for stroke.^12^ By learning from these mistakes, scientists are working to develop techniques for nondestructive longitudinal imaging of the biodistribution of drugs, exemplified by radiolabeling of drug molecules for subsequent positron emission tomography imaging.^13^ Some drugs are being developed as theranostic agents where a complex therapeutic system is developed to fulfill both therapeutic and diagnostic purposes. Such imaging strategy provides the necessary information to improve protocols for an increased accumulation of these drugs in the brain and ultimately better therapies.

However, the entire field is still at its infancy, and the clinical impact of novel neuroprotective and neurorestorative approaches remains limited and modest. Additional research efforts, both preclinically and clinically, are required to increase this impact and generate strong evidence for the efficacy of new neuroprotective approaches in large‐scale clinical trials. This is likely to come in part from continued back‐translation of what we have learnt clinically as well as future paralleled preclinical and clinical studies to gain better mechanistic insight. Nevertheless, it is not unreasonable to claim that there was rarely a better time to enter the neuroprotection and neurorestoration fields, and to work in translational neuroscience. What is still missing though is a platform to bundle and share the knowledge generated in the field. Cooperating with Wiley, a renowned scientific publisher, and partnering with the Chinese Medical Association we intend to fill this gap by launching the new journal *Neuroprotection*.

*Neuroprotection* publishes original research and reviews reporting recent advances and breakthroughs in the fields of neuroprotection and nervous system repair. The journal is not restricted to any particular condition but focuses on the entire spectrum of central and peripheral nervous system diseases. *Neuroprotection* is an open‐access journal and has a strong interdisciplinary approach including areas such as neurobiology, neuropharmacology, neurology, neurosurgery, neuroimaging, neurorehabilitation, and basic as well as translational neuroscience. The journal's audience, including basic researchers, translational scientists and clinicians, reflects this interdisciplinarity. *Neuroprotection* is dedicated to a rigorous peer‐review process and outstanding quality of publications from the start. As a welcoming gift to the neuroscience community, but also to facilitate a large number of submissions to select the very best manuscripts for publication, we decided to waive all article processing and publication fees during the first 2 years after the journal's launch. *Neuroprotection* is now accepting manuscripts, applying a free format submission policy. This means that authors can submit articles in their format of choice, and particular formatting requirements do not need to be met at this stage. This makes the submission process smoother and less time‐consuming. The journal's website can be found at https://onlinelibrary.wiley.com/journal/2770730x.

Please join us on an amazing scientific journey into neuroprotection in research and clinics, and submit your manuscript to *Neuroprotection*! We do look forward to your contribution!

## AUTHOR CONTRIBUTIONS

All authors listed have made a substantial, direct, and intellectual contribution to the work and approved it for publication.

## CONFLICTS OF INTEREST

The authors declare that they have no known competing financial interests or personal relationships that could have appeared to influence the work reported in this paper. Xunming Ji, Piotr Walczak, and Johannes Boltze are Co‐Editors in Chief of Neuroprotection, Shen Li is the journal's executive editor. All other authors are members of the editorial board. They were blinded from reviewing or making decisions on the manuscript. The article was subject to the journal's standard procedures, with peer review handled independently of this Editorial Board member and their research groups. Piotr Walczak is a founder and holds equity IntraArt, LLC. Ti‐com, LLC.

## ETHICS STATEMENT

This article does not contain any studies with human participants or animals performed by any of the authors.

## Data Availability

All the data are included in the manuscripts.
